# Short-term high glucose culture potentiates pancreatic beta cell function

**DOI:** 10.1038/s41598-018-31325-5

**Published:** 2018-08-30

**Authors:** Eduardo Rebelato, Laila R. Santos, Angelo R. Carpinelli, Patrik Rorsman, Fernando Abdulkader

**Affiliations:** 1 Department of Biophysics, Federal University of Sao Paulo, Sao Paulo, Brazil; 2 Institute for Vascular Signalling, Centre for Molecular Medicine, Goethe University, Frankfurt, Germany; 3 Department of Physiology and Biophysics, Institute of Biomedical Sciences, University of Sao Paulo, Sao Paulo, Brazil; 4 Oxford Centre for Diabetes, Endocrinology and Metabolism, Oxford University, Oxford, United Kingdom

## Abstract

The exposure of pancreatic islets to high glucose is believed to be one of the causal factors of the progressive lowering of insulin secretion in the development of type 2 diabetes. The progression of beta cell failure to type 2 diabetes is preceded by an early positive increase in the insulin secretory response to glucose, which is only later followed by a loss in the secretion capacity of pancreatic islets. Here we have investigated the electrophysiological mechanisms underlying the early glucose-mediated gain of function. Rodent pancreatic islets or dispersed islet cells were cultured in medium containing either 5.6 (control) or 16.7 (high-glucose) mM glucose for 24 h after isolation. Glucose-stimulated insulin secretion was enhanced in a concentration-dependent manner in high glucose-cultured islets. This was associated with a positive effect on beta cell exocytotic capacity, a lower basal K_ATP_ conductance and a higher glucose sensitivity to fire action potentials. Despite no changes in voltage-gated Ca^2+^ currents were observed in voltage-clamp experiments, the [Ca^2+^]_I_ responses to glucose were drastically increased in high glucose-cultured cells. Of note, voltage-dependent K^+^ currents were decreased and their activation was shifted to more depolarized potentials by high-glucose culture. This decrease in voltage-dependent K^+^ channel (Kv) current may be responsible for the elevated [Ca^2+^]_I_ response to metabolism-dependent and independent stimuli, associated with more depolarized membrane potentials with lower amplitude oscillations in high glucose-cultured beta cells. Overall these results show that beta cells improve their response to acute challenges after short-term culture with high glucose by a mechanism that involves modulation not only of metabolism but also of ion fluxes and exocytosis, in which Kv activity appears as an important regulator.

## Introduction

A hallmark of type 2 diabetes is a reduced insulin secretory capacity. When combined with insulin resistance, this results in impaired glucose tolerance and diabetes. However, prior to the onset of diabetes, the beta cells compensate for insulin resistance by increased insulin secretion.

Although normal fasting plasma glycaemia is observed in glucose intolerance, the postprandial blood glucose and the corresponding insulin response are elevated in this period. In this scenario, hyperinsulinemia is characterized as a beta cell response to the intermittent exposure of pancreatic beta cells to high glucose levels as a consequence of insulin resistance.

Classically, *in vitro* long-term exposure of pancreatic beta cells to high glucose causes defects in insulin secretory capacity^1^. Ten days of high glucose exposure increases basal insulin secretion and basal levels of intracellular Ca^2+^, but impairs the maximum insulin secretion capacity^2^. Although previous studies have shown that long-term exposure to nutrients enhance beta cell metabolism, with a major effect on glucokinase activity and mitochondrial function^3^, the general response of these cells to hyperglycaemia culminates with impairment in cell function^4^. However, an *in vivo* study with partially pancreatectomized rats with moderate hyperglycaemia showed that, even after two weeks, pancreatic beta cells presented a leftwards shift in the dose-response curve of glucose-stimulated insulin secretion (GSIS)^5^. Thus, the effect of elevated glucose on beta cell function appears to be dependent on the combination of the glucose level and the duration of exposure.

Conversely, in short-term exposure, while there is some opposite evidence^6–8^, it has been shown that the exposure of beta cells to elevated glucose levels can promote an improvement in cell function^9–11^. This raises the interesting possibility that the hyperinsulinaemia in prediabetic individuals may not just be an insulin secretory response to overcome changes in glycaemia imposed by insulin resistance. Instead, hyperinsulinaemia appears also to be a consequence of beta cell adaptive responses to moderate hyperglycaemia. Here we have investigated functional changes in beta cells after 24 h exposure to control (5.6 mM) and high (16.7 mM) glucose using an *in vitro* model of glucose intolerance.

## Results

### High-glucose culture potentiates insulin secretory response

**Figure 1:**
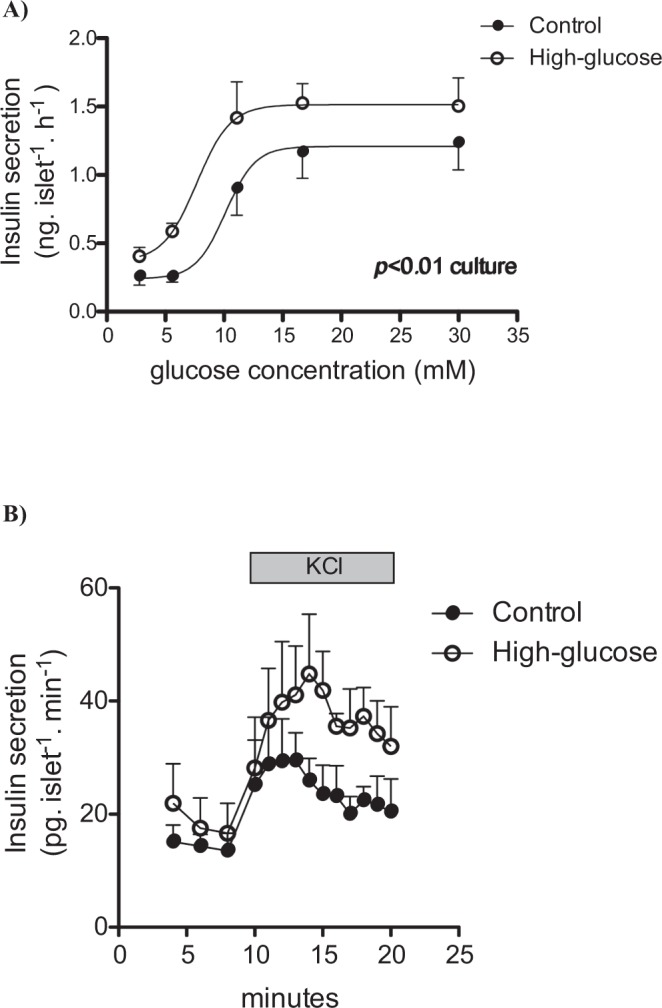
(**A**) Static insulin secretion response to different glucose concentrations (n = 3–7). (**B**) Dynamic insulin secretion response to glucose (16.7 mM) or KCl (35 mM) of pancreatic islets cultured at 5.6 or 16.7 mM glucose for 24 h (n = 2). *p* < 0.01 *vs* culture condition.

High glucose-cultured islets secreted insulin at a higher rate than those cultured at 5.6 mM glucose. In control islets insulin secretion was initiated at glucose concentrations >5.6 mM (EC_50_ 10.13 mM). In islets cultured at 16.7 mM glucose, basal insulin secretion was 53% higher and the GSIS already started at 5.6 mM (EC_50_ 7.67 mM). High-glucose cultured islets presented increased insulin secretion at all glucose concentrations (Fig. 1A).

Additionally, in dynamic secretion experiments using metabolism-independent depolarization (35 mM KCl) islets showed an increased secretory response in both first and second phases of insulin secretion in high-glucose compared to control islets (Fig. 1B).

### High-glucose potentiates exocytosis

**Figure 2:**
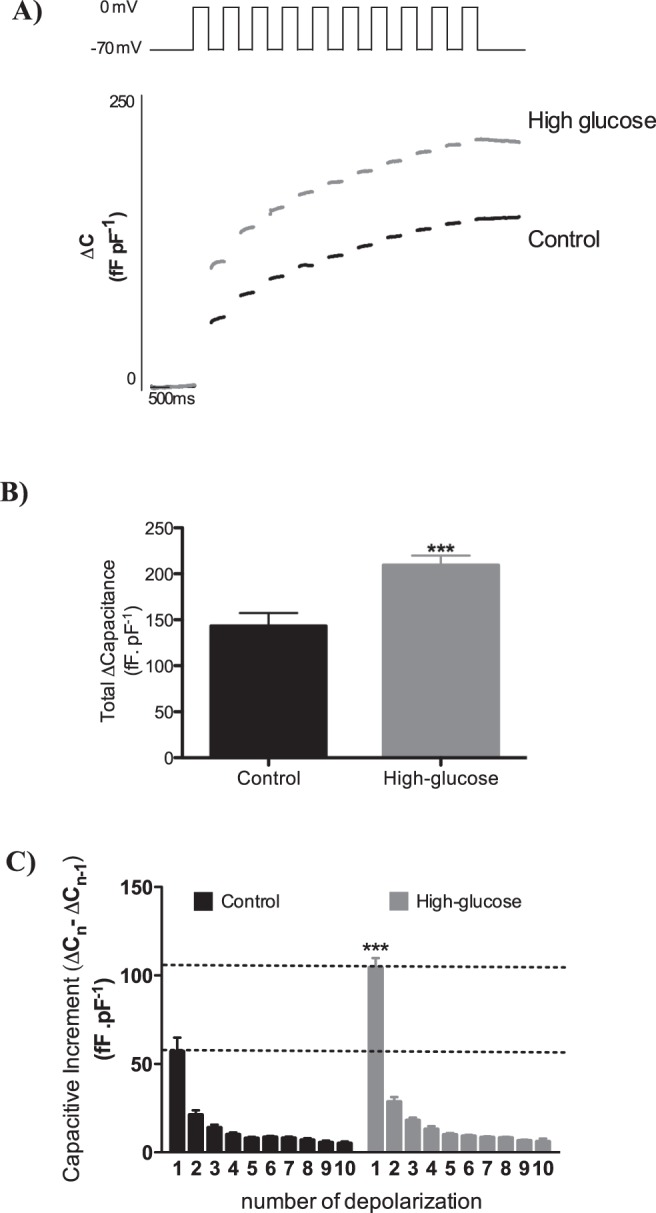
(**A**) Averaged traces of capacitance measurements of single cell exocytosis in pancreatic islet cells cultured for 24 h at 5.6 (n = 37) or 16.7 mM glucose (n = 30). (**B**) Total increase in capacitance increment of the recordings presented in A. (**C**) Capacitance increment in each pulse of depolarization in the recordings presented in A. ***p ≤ 0.001 *vs* culture condition.

The finding that insulin secretion was enhanced in high glucose-cultured islets regardless of the glucose concentration and whether glucose or high-K^+^ was used as the stimulus, suggests that there might be a direct effect on exocytosis. We tested this by capacitance measurements using the patch-clamp technique in standard whole-cell configuration, in which the intracellular environment is determined by the pipette solution and so is the same in both cell conditions. In response to a train of ten depolarizations (500 ms) from the holding potential (−70 mV) to 0 mV, exocytosis was enhanced in high-glucose compared to control beta cells (Fig. 2A,B). We also plotted the capacitance increase for the tandem individual depolarizations during the train. Interestingly, the main effect on the capacitive response to the depolarization occurred in the first pulse (Fig. 2C).

### Triggering of electrical activity is shifted to lower glucose levels in high-glucose beta cells

To evaluate the possible changes in beta cell electrical activity in response to glucose, the membrane potential of beta cells in intact pancreatic islets was recorded during perifusion with increasing glucose concentrations.

**Figure 3:**
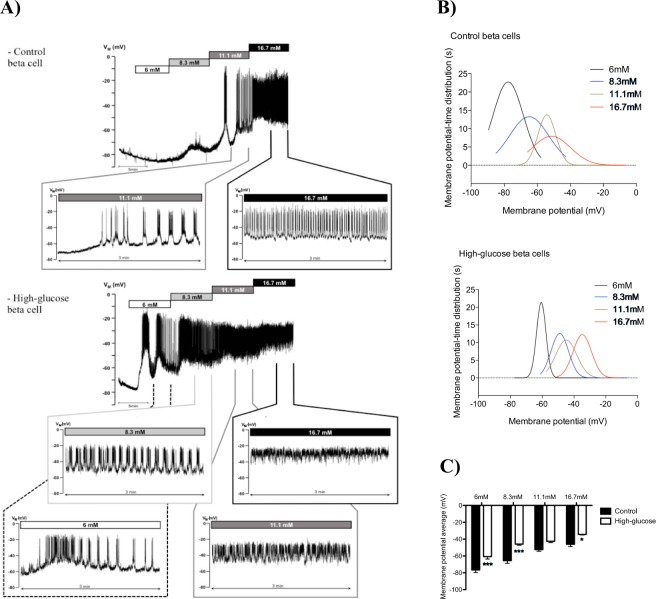
(**A**) Representative recordings of electrical activity of beta cells in intact pancreatic islets acutely exposed to increasing glucose concentrations. Insets in panel A show the last 3 minutes of cell excitability triggered by different glucose concentrations. (**B**) Non-linear fit of membrane potential-time distribution and (**C**) average of membrane potential at the last 3 minutes of the acute glucose exposure of pancreatic islet cells cultured for 24 h at 5.6 or 16.7 mM glucose (n = 3–5 cells for each culture condition). *p ≤ 0.05 and ***p ≤ 0.001 *vs* culture condition.

In control beta cells the membrane potential at 1 mM glucose was ~ −77 mV. Increasing glucose to 6 mM had no effect, but a shift in membrane potential to ~ −65 mV occurred at 8.3 mM glucose, while no regenerative electrical activity was observed. Action potential firing was initiated at 11 mM glucose and consisted of bursts of action potentials, with a higher frequency at 16.7 mM. The high glucose-cultured beta cells responded rather differently. In these cells the resting membrane potential at 1 mM glucose was ~ −71 mV and the response to all glucose concentrations was enhanced. Resting potential changes to ~ −60 mV were already observed at 6 mM glucose. This indicates that the sensitivity of high-glucose islets to acute glucose exposure was shifted to lower glucose levels, as in all high-glucose beta cells recorded the firing of action potentials started between 6 and 8.3 mM glucose. (Fig. 3).

Together with this higher sensitivity to acute glucose, it was also possible to observe a strong depolarizing effect in those cells. Insets of Fig. 3A display representative traces of the depolarizing pattern in response to acute exposure to different glucose concentrations. In the presence of both 11.1 or 16.7 mM glucose, high-glucose beta cells remain most of the time at a more depolarized basal potential (Fig. 3A).

A non-linear fit of the membrane potential-time distribution (Fig. 3B) and the average membrane potential (Fig. 3C) in response to acute glucose stimulus are shown for control and high-glucose beta cells. In these figures it is possible to observe a shift to a more depolarized membrane potential in response to all glucose concentrations in high-glucose beta cells compared to control.

Thus, in the presence of glucose concentrations above 6 mM, high glucose-cultured beta cells spend more time in depolarized potentials than control cells and their basal membrane potential is steadily depolarized following the increment in glucose concentration (Fig. 3C).

### Basal K_ATP_ channel activity is reduced in high glucose-cultured beta cells

**Figure 4:**
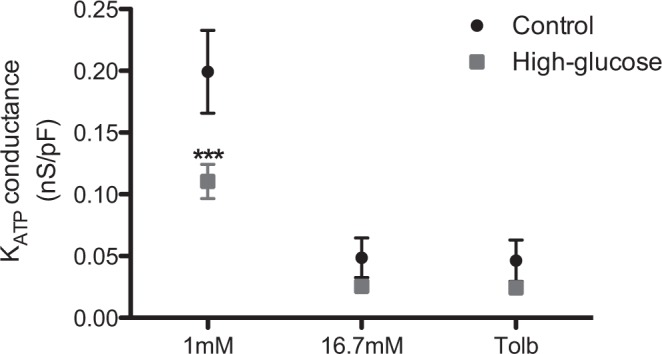
Basal membrane conductance at different stimuli (n = 8–13 cells). ***p ≤ 0.001 *vs* culture condition.

ATP-regulated K^+^ channels (K_ATP_) represent the glucose-regulated membrane conductance of the beta cell. To understand the increased excitability of high glucose-cultured beta cells, we measured K_ATP_ conductance using the perforated patch whole-cell technique in which cellular metabolism is preserved (Fig. 4). At 1 mM glucose, the resting K_ATP_ conductance in control beta cells was 0.2 nS. pF^−1^. Increasing glucose reduced the whole-cell conductance by 76 or 77% due to exposure to 16.7 mM or tolbutamide, respectively. In high glucose-cultured beta cells the K_ATP_ conductance observed in response to high glucose or tolbutamide was similar to that in control cells. However, it was observed that at 1 mM glucose high glucose-cultured cells already presented a lower K_ATP_ conductance, 0.1 nS. pF^−1^.

### Intracellular Ca^2+^ handling, but not Ca^2+^ channels, is affected by high-glucose culture

**Figure 5:**
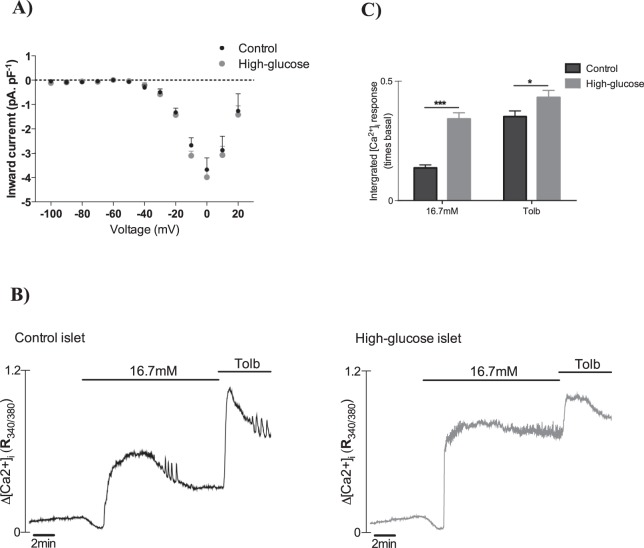
(**A**) Steady-state Ca^2+^ current-voltage relationship in beta cells cultivated for 24 h at 5.6 (n = 26 cells) or 16.7 mM glucose (n = 32 cells). (**B**) Representative recordings of calcium inflow in response to acute glucose and tolbutamide of intact pancreatic islets cultivated for 24 h at 5.6 or 16.7 mM glucose. (**C**) Integrated Ca^2+^ response from figures B (n = 11–14 islet). *p ≤ 0.05; ***p ≤ 0.001.

The effect of high-glucose culture on the Ca^2+^ current was analysed by standard patch-clamp in voltage-clamp mode. Beta cells were submitted to changes in membrane potential from −100 to +20 mV in steps of 10 mV. In these experiments, the glucose-culture condition did not affect the Ca^2+^ currents triggered by changes in membrane potential (Fig. 5A).

As intrinsic voltage-dependent Ca^2+^ channel (Ca_v_) activity seems not to be affected by the culture conditions, we sought to evaluate the changes in [Ca^2+^]_I_ in the evolution of free course changes in membrane potential triggered by glucose and tolbutamide. In control islets, basal [Ca^2+^]_I_ was low and increasing glucose elicited a triphasic response consisting of an initial lowering followed by a peak in [Ca^2+^]_I_ that declined to a new steady-state plateau, in which discrete oscillations were observed (presumably reflecting bursts of action potentials synchronised across the islet) (Fig. 5B). In high glucose-cultured islets the initial increase was much larger (*p* ≤ 0.001) and (unlike what was seen in control islets) maintained throughout the glucose application. Tolbutamide had a marked effect to sustain intracellular Ca^2+^ at high levels in high glucose-cultured islets (Fig. 5B). Figure 5C compares the integrated [Ca^2+^]_I_ response between control and high glucose-cultured islets in response to 16.7 mM glucose and tolbutamide. It shows that the differences in [Ca^2+^]_I_ were mostly observed in response to 16.7 mM glucose, when metabolism was involved.

### High-glucose culture reduces the action potential repolarizing currents

**Figure 6:**
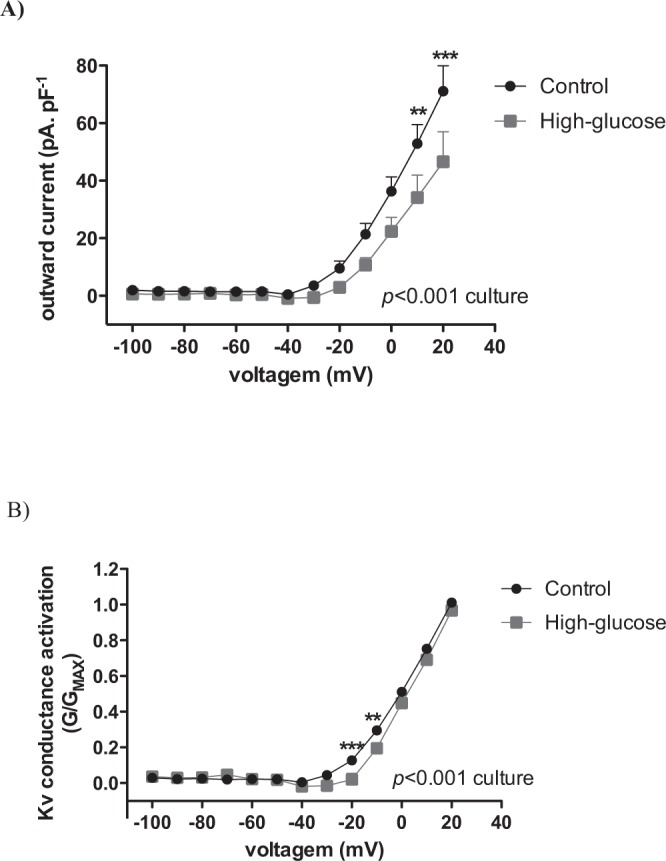
(**A**) Voltage-dependent potassium current in pancreatic beta cells cultivated for 24 h at 5.6 or 16.7 mM glucose. (**B**) Conductance-voltage relationships of Kv currents (n = 9). Two-way Anova: glucose *p* ≤ 0.001; voltage *p* ≤ 0.001; interaction *p* ≤ 0.05. Bonferroni’s post-test **p ≤ 0.01 and ***p ≤ 0.001 *vs* culture condition.

To evaluate the involvement of active repolarizing currents in the high [Ca^2+^]_I_ response and basal membrane potential observed in high glucose-cultured beta cells, the voltage-dependent K^+^ current was analysed. In control cells the activation of voltage-dependent K^+^ channels (Kv) started at −30 mV with a current of 3.5 pA. pF^−1^, reaching 71 pA. pF^−1^ at +20 mV. In high glucose-cultured cells the activation of these channels was observed at −20 mV with a current of 2.9 pA. pF^−1^, reaching 46 pA. pF^−1^ at +20 mV. Thus, the high glucose culture significantly reduced the voltage-dependent K^+^ current (Fig. 6A) and promoted a right-shift in the Kv activation curve (Fig. 6B).

## Discussion

Here we have studied the effects of 24 h *in vitro* culture of rodent beta cells and islets with high glucose, which recapitulates the early gain-of-function, marked by hyperinsulinaemia, during the development of glucose intolerance. This enhanced GSIS was shown to involve changes in the sensitivity and the profile of membrane potential electrical activity elicited by increasing glucose concentrations^11^, as well as increased exocytosis efficiency and reduced voltage-dependent K^+^ conductance.

Most experimental studies on the progression of glucose intolerance towards type 2 diabetes have focused on the associated beta cell dysfunction, rather than the early gain-of-function of beta cells that sustain whole-body glucose homeostasis via increased insulin secretion. *In vivo*, the gain-of-function of pancreatic beta cells in response to glucose intolerance was reported to occur for instance in ob/ob mice^12^. *In vitro*, positive effects of high-glucose culture on beta cell function were previously reported to occur by the potentiation of metabolic rates^3^ and they were also observed as an increment in beta cell sensitivity to glucose in islets cultured for longer periods, in which such effect was suggested to be a consequence of a high activity of the triggering and amplifying pathways^2^. However, contrary to previous reports^6–8^, our results and others’^9–11^ demonstrate an increase in beta cell sensitivity to glucose (Figs 1 and 3) associated with a higher maximum secretory response to glucose after culture in high-glucose for periods not longer than 24 h (Fig. 1). Thus, we were able to observe that the positive changes previously demonstrated to occur as early as within 12 h culture in beta cells at high-glucose^11^ are still preserved at 24 h, and they were associated with an increase in the secretory capacity (Fig. 2).

The increased sensitivity to glucose can at least in part be attributed to the decreased basal K_ATP_ conductance, similarly to what has also been reported by Glynn *et al*.^11^. Potassium membrane permeability can be further regulated by metabolic signals other than ATP. For instance low glucose, via AMPK, was reported to increase K_ATP_ trafficking and insertion into the plasma membrane, inducing cell hyperpolarization^13^. In this sense, high availability of glucose during hyperglycaemic conditions may decrease K_ATP_ density at the plasma membrane, by an inhibition of AMPK signalling, causing reduction in K^+^ leak currents. This lower K_ATP_ conductance observed in high glucose-cultured beta cells seems to require only a small additional variation in intracellular ATP levels to bring the membrane potential to the opening threshold of voltage-dependent Ca^2+^ channels.

However, additional mechanisms other than K_ATP_ should be involved in the hyper-secretion observed in high glucose-cultured beta cells in response to 16.7 mM or higher glucose concentrations, in which K^+^ leak conductances would be indistinguishable (Fig. 4). The first candidate mechanism is the activity of voltage-dependent Ca^2+^ channels that can be positively modulated via cAMP-dependent phosphorylation^14^, which could be increased in high-glucose scenarios.

Contrary to our initial hypothesis, no increased voltage-dependent Ca^2+^ conductance was observed in the high glucose-cultured beta cells compared to controls (Fig. 5A). However, even in the absence of differences in Ca^2+^ currents the immediate depolarization-evoked exocytosis was substantially increased in high glucose beta cells (Fig. 2), which suggests an increased efficiency of the exocytotic machinery.

Despite the absence of differences in standard whole-cell Ca^2+^ conductance, when the intracellular Ca^2+^ concentration was evaluated, striking differences were observed between islets from the two culture conditions. Not only was the amplitude of the response significantly increased in high-glucose islets (Fig. 5C), but also the pattern of the response was different (Fig. 5B). Although it has been shown that the major contributors to the increase in [Ca^2+^]_I_ are the L-type channels in beta cells^15,16^, a role of the endoplasmic reticulum (ER) in calcium handling cannot be rule out^17^. Thus, it is possible that in metabolically preserved beta-cells (i.e. not in standard whole-cell mode), the higher [Ca^2+^]_I_ observed in high-glucose cells (Fig. 5B, C) may be due to changes in intracellular metabolic signals^18^ that can affect either voltage-dependent Ca^2+^ channels^14^ or to the participation of ER calcium.

Additionally, the glucose-stimulated [Ca^2+^]_I_ response was somewhat clamped at high levels in the high-glucose islets, while it was free to rhythmically oscillate in control islets. This correlates with the electrical activity pattern detected in cells from each culture condition, i.e. membrane potentials with lower amplitude oscillations but starting from a significantly more depolarized baseline in the high glucose-cultured beta cells at the acute 16.7 mM glucose stimuli (Fig. 3) when K_ATP_ channels are totally closed (Fig. 4). This additional baseline depolarization in high-glucose beta cells may thus be a consequence of decreased voltage-dependent K^+^ currents (Fig. 6A). In this sense, the reduction in voltage-activated K^+^ currents (Fig. 6A) and the delay in the K^+^ conductance activation (Fig. 6B) caused by high-glucose culture impairs the membrane repolarizing capacity and can contribute to the elevated [Ca^2+^]_I_ response. This can also be further suggested by the difference in [Ca^2+^]_I_ response to 1 mM glucose (low cellular metabolic activity) plus the K_ATP_ antagonist, tolbutamide (Fig. 5C). Thus, the strong depolarizing pattern observed in high-glucose beta cells could allow the voltage-dependent Ca^2+^ channels to conduct for longer periods without being deactivated by repolarization.

Voltage-dependent K^+^ channels have been shown to be expressed and functional in both rodent and human beta cells^19^ (for a review, see MacDonald & Wheeler, 2003). Two Kv isoforms are importantly expressed in beta cells, whose phenotype is of delayed rectifiers and their activity was identified to decrease GSIS^20^, as it would be expected from their hyperpolarizing current.

The Kv current was previously reported to be increased after 3 to 5 days in culture at 28 mM glucose, which was paralleled by increased protein expression of the isoform Kv2.1^21^. Although we have not investigated the identity of Kv currents, we found at an earlier stage of culture (24 h) at 16.7 mM glucose a decrease, rather than an increase, in Kv currents and a delay in voltage activation of Kv conductances. A possible mechanism behind this effect could be due to metabolic changes, via increases in NAD(P)H that has been reported to favour Kv2 inhibition^22^.

The mechanisms responsible for this early gain in function of beta cells via the exacerbated calcium response due to the impairment in repolarization may trigger the later defects in beta cells observed during the progression of their dysfunction. Continuously high-calcium levels can activate the carbohydrate response element-binding protein (ChREBP)^23^, which is known to increase its activity in a high-glucose environment and to play a pivotal role in beta cell glucotoxicity^24^.

Thus, with the understanding of the early gain in function of beta cells one can shed light into the mechanisms that may lead to their dysfunction in the context of hyperglycaemia. Specifically, the early beneficial changes in calcium handling during the gain phase may evolve to dysfunction in the progression of glucose intolerance towards overt type 2 diabetes.

## Methods

### Pancreatic islet isolation and culture

Pancreatic islets were isolated by collagenase digestion^25^ from Wistar rats for static insulin secretion, calcium current and capacitance measurements or from NMRI mice for all other experiments. All experiments were approved either by the Animal in Science Regulation Unit of the UK Home Office (licence number P0FA927F8) and the IACUC of the Institute of Biomedical Sciences of the University of São Paulo (licence number 159/2012). The experiments were conducted in accordance with the UK Animals Scientific Procedures Act (1986) and University of Oxford ethical guidelines and the guidelines for laboratory animal use established by the Brazilian federal law on animal care and use. The islets or dispersed cells were then cultured in a humidified atmosphere containing 5% CO_2_ in complete RPMI 1640 medium supplemented with 10% foetal calf serum, 100 U/mL penicillin, 100 μg/mL streptomycin, with 5.6 (control) or 16.7 (high glucose) mM glucose for 24 h prior to the experiments.

The electrical activity recordings were performed in single beta cells in intact pancreatic islets. For the measurement of cell capacitance, K_ATP_ conductance, Ca^2+^ and K^+^ voltage-dependent currents, the islets cells were dispersed using trypsin and pleated onto adherent dishes (Sarstedt, Germany) prior to culture.

### Insulin secretion

For static experiments, after 24 h culture, batches of 5 islets were pre-incubated at 37 **°**C in 500 μL of Krebs-Henseleit buffer containing (in mM) 115 NaCl, 5 KCl, 1 CaCl_2_, 1 Mg Cl_2_, 24 NaHCO_3_, pH 7.4, supplemented with albumin (0.2%) and 5.6 mM glucose for 30 min. Then, the islets were incubated for 1 hour in the same buffer with different glucose concentrations.

For dynamic experiments, after 24 h culture, batches of 50 islets were transferred to a perifusion chamber. The perifusion experiments were performed at 37 °C with Krebs-Henseleit buffer containing albumin (0.2%) and 2.8 mM glucose. For the perifusion with Krebs-Henseleit containing KCl (35 mM), NaCl was lowered to maintain iso-osmolarity. The islets were perifused at a constant flow rate of 0.5 mL/min and the perifusate was collected every minute and the insulin was later assayed by radioimmunoassay.

### Exocytosis and whole-cell Ca^2+^ current

After 24 h, the dishes with the dispersed islet cells were placed on the stage of an inverted microscope and perifused with extracellular solution consisting of (mM) 118 NaCl, 20 TEACl, 5.6 KCl, 2.6 CaCl_2_, 1.2 MgCl_2_, 5 HEPES (pH 7.4 with NaOH) and either 5.6 or 16.7 glucose. As no difference was observed between cells from the same culture treatment for both extracellular glucose solutions (data not shown), the results are presented here combined. Exocytosis, evaluated by membrane capacitance, and the Ca^2+^ current were recorded using an EPC-10 amplifier (HEKA, Lamprecht, Germany) in standard whole-cell configuration and voltage-clamp mode using borosilicate glass pipettes (pipette solution (mM): 129 CsOH, 125 Glutamic acid, 20 CsCl, 10 NaCl, 1 MgCl_2_, 5 HEPES, 0.05 EGTA, 3 MgATP, 0.1 cAMP, pH 7.2). All the experiments were performed at 32–33 °C. To elicit exocytosis, a train of 10 depolarizing (0 mV) pulses was applied, each pulse with 0.5 s duration at 1 Hz. For the inward calcium current analyses, current-voltage (I–V) curves were obtained by application of 100 ms depolarizations to membrane potentials between −100 to +20 mV (in 10 mV steps).

### Electrical activity, K_ATP_ conductance and voltage-dependent K^+^ current recordings

Beta cells were perifused with extracellular solution consisting of (mM) 140 NaCl, 3.6 KCl, 1.5 CaCl_2_, 0.5 NaH_2_PO_4_, 0.5 MgSO_4_, 5 HEPES, 2 NaHCO_3_ and pH 7.4, containing different glucose concentrations. The experiments were recorded using an EPC-10 amplifier (HEKA, Lamprecht, Germany) in perforated whole-cell configuration in current-clamp or voltage-clamp mode, as appropriate, using a glass pipette (pipette solution (mM): 10 NaCl, 10 KCl, 76 K_2_SO_4_, 1 MgCl_2_, 5 HEPES, pH 7.35, amphotericin B 60 μg/mL). All the experiments were performed at 32–33 °C. The K_ATP_ conductance was derived from the leak current observed in response to a (±) 5 mV square pulse from a holding potential of −70 mV (close to the beta cell resting potential). Voltage-dependent K^+^ currents were determined in the perforated patch configuration by an I–V curve with 60 ms depolarizations to membrane potentials between −100 to +20 mV (in 10 mV steps). The activation of Kv conductance was calculated as G/G_MAX_ assuming a Hodgkin-Huxley model for voltage-activated K^+^ conductances based on the Goldman-Hodgkin-Katz flux equation. For the quantification of the electrical activity, current-clamp data were analysed for the last 3 min of recording at each glucose stimulus. Frequency histograms for each mV membrane potential were obtained and pooled between cells in the same experimental condition. Afterwards, Gaussian distributions were fitted to the pooled data of each acute glucose stimulus.

### Imaging of intracellular Ca^2+^

Islets were loaded at room temperature with 6 μM Fura-2 AM (Invitrogen, Paisley, U.K.) plus 0.02% pluronic acid. After loading, islets were placed on the stage of an inverted microscope and perifused with an extracellular solution (mM) 138 NaCl, 4.8 KCl, 2.6 CaCl_2_, 0.5 NaH_2_PO_4_, 0.5 MgSO_4_, 5 HEPES, 2 NaHCO_3_ (pH 7.4 with NaOH) containing different glucose concentrations (1 or 16.7 mM), or at 1 mM glucose plus 100 μM tolbutamide. Intracellular Ca^2+^ was ratiometricaly recorded using a dual-wavelength fluorescence microscopy system (Photon Technology International, Monmouth Junction, NJ). For probe excitation, 340 and 380 nm wavelengths were used, and the emitted fluorescence was captured at 510 nm. All the experiments were performed at 35–37 °C. The integrated Ca^2+^ response was calculated as the average response for each stimulus.

### Statistical analysis

Results are presented as means ± SEM. Statistical significance was determined by Two-way ANOVA with Bonferroni’s post-test or by Student’s t test, as appropriate. Differences were considered significant for *p* ≤ 0.05.
